# Plant Root Secretion Alleviates Carbamate-Induced Molecular Alterations of Dissolved Organic Matter

**DOI:** 10.3390/toxics12090654

**Published:** 2024-09-05

**Authors:** Zihan Niu, Chao Chen, Qijun Ruan, Yingming Duan, Shuqin Liu, Da Chen

**Affiliations:** 1 Guangdong Key Laboratory of Environmental Pollution and Health, School of Environment and Climate, Jinan University, Guangzhou 511443, China; 2 Guangdong Provincial Key Laboratory of Chemical Measurement and Emergency Test Technology, Institute of Analysis, Guangdong Academy of Sciences (China National Analytical Center Guangzhou), Guangzhou 510070, China; 3 China College of Resources and Environmental Engineering, Guizhou University, Guiyang 550025, China

**Keywords:** carbamate exposure, in vivo analysis, dissolved organic matter, plant root secretion, Fourier transform ion cyclotron resonance mass spectrometry, solid phase microextraction

## Abstract

Studying the interaction between pesticide contamination in the plant system and the dissolved organic matter (DOM) composition is important to understand the impact of pesticides and plants on the ecological function of DOM. The present study investigated the effects of DOM on the bioaccumulation and biotransformation of carbamates in plants, carbamate exposure on DOM composition, and plant root secretion on the interaction between DOM and carbamates. The concentrations of carbamates and their metabolites in living cabbage plants were continuously tracked through an in vivo analytical method. The presence of DOM was found to reduce the highest bioconcentrations and shorten the time it took to reach the highest bioaccumulated amounts of isoprocarb and carbofuran in plants, while it showed no significant effect on the uptake behavior of carbaryl. DOM profiling results indicated that carbamate exposure substantially decreased the number and molecular diversity of DOM. Notably, plant root secretion alleviated carbamate-induced DOM molecular alterations by inducing a higher turnover rate of DOM compared to that in the uncontaminated group, highlighting the role of plants in mitigating the effects of exogenous pesticide exposure on DOM composition and maintaining DOM molecular homeostasis.

## 1. Introduction

Dissolved organic matter (DOM) exists ubiquitously in the environment [1,2,3] and the interactions between DOM and environmental pollutants have attracted considerable attention [4,5,6,7]. DOM plays a crucial role in the biogeochemical processes of pollutants. Increasing evidence has suggested that the chemical composition of DOM considerably affects the migration [8,9], transformation [4], and bioavailability [4,7,10,11] of pollutants in the environment, impacting pollutant mitigation. DOM has been proven to affect the migration of carbamazepine and sulfamethoxazole [8] and alter the photodegradation behavior of organic pollutants, such as pesticides, endocrine disruptors, and antibiotics [12,13]. Additionally, it has been reported that DOM regulates the bioaccumulation and phytotoxicity of silver nanoparticles to Lemna minor7 and influences the translocation and transformation of chlorinated polyfluoroalkyl ether sulfonate in wheat [4].

Meanwhile, environmental pollutants may, in turn, alter the normal composition of DOM, affecting its ecological functions [5,14]. Sun et al. investigated the effects of several biodegradable and non-biodegradable microplastics on DOM composition in black and yellow soils and found that plastic-leached DOM could trigger changes in DOM in different soils [5]. Sheridan et al. discovered that in addition to altering the DOM composition in the environment, environmentally relevant concentrations of plastic leachate can increase bacterial biomass acquisition in lake surface waters by 2.29 times [14]. Given that the composition of DOM determines its environmental function [15,16], studying the influence of pollutants on its molecular composition is of considerable importance in understanding the role of pollutants in the geochemical process of DOM. However, although various studies have investigated the impact of DOM on the environmental behavior of pollutants, relatively few have addressed the influence of pollutants on DOM which primarily focused on the effects of plastic-leached organic components on changes in DOM composition and ecological functions. The effects of other pollutants on environmental DOM remain largely unknown.

Pesticides have been widely used since the 1940s to prevent agricultural losses due to diseases caused by insects and weeds. Currently, the annual global consumption of chemical pesticides reaches millions of tons [17,18,19]. Carbamate pesticides are widely used in forestry, gardening, agriculture, and therapeutic pharmaceuticals because of their relatively low bioaccumulation and environmental persistence [20,21,22]. However, some carbamates, such as carbofuran, may easily damage the biological properties of soil and the enzymatic activities of plants, as well as cause geno-, neuro-, and reproductive toxicities as well as dermal skin problems in humans [22,23]. Carbamate consumption has exceeded that of pyrethroids, organophosphates, and organochlorines for agricultural or indoor purposes, although the United States, Europe, and China partially restricted or banned the use of some carbamates in agriculture from 2006 to 2017 [24,25,26]. The potential health risks and adverse effects of carbamate pesticides have attracted considerable attention in recent years [22,24]. Fresh vegetables are the main receptors of carbamates and are a source of pesticides for non-occupational exposure. Given the widespread use of carbamate pesticides, it is important to examine the effects of DOM on the bioaccumulation and biotransformation of carbamates in plant tissues, as well as how carbamates influence environmental DOM composition. Additionally, while it is known that plant root secretion affects DOM composition [27], its impact on the interactions between exogenous pollutants and DOM remains unclear.

Therefore, the objectives of this study were as follows: (1) to investigate the effects of DOM on bioaccumulation and biotransformation of carbamates in plants; (2) to determine the impact of carbamates on DOM composition; and (3) to explore the role of plant root secretions in the interaction between DOM and carbamates. A 21-day hydroponic experiment was conducted using cabbage plants as the test organism and humic acid (HA) as a model of soil DOM. Carbamate concentrations in the plants and DOM compositions in the system were continuously sampled and analyzed throughout the 21-day experiment. The DOM compositions were analyzed using ultra-high-resolution Fourier transform ion cyclotron resonance mass spectrometry (FT-ICR-MS), which allowed detailed profiling of DOM at the molecular level [28,29,30,31]. A non-destructive in vivo analytical method proposed in our previous work was used for the long-term monitoring of carbamates and carbamate metabolites in plant tissues without damaging the living plants [32]. This method eliminated the need for plant sacrifice and prevented the disturbance to DOM caused by traditional lethal sampling methods.

## 2. Materials and Methods

### 2.1. Chemicals and Reagents

Standards of carbamates and their metabolites, i.e., carbofuran, carbofuran phenol, *o*-cumenol, and 1-naphthalenol were purchased from Dr. Ehrenstorfer GmbH (Augsburg, Germany) while isoprocarb and carbaryl were bought from Aladdin (Shanghai, China). The PPL solid-phase extraction (SPE) columns (Bond Elut PPL-200 mg 3 mL) were bought from Agilent (Santa Clara, CA, USA). Humic Acid (HA) was purchased from Sigma-Aldrich (St. Louis, MO, USA).

### 2.2. Exposure Experiments

This study focused on investigating the effects of DOM on the bioaccumulation and biotransformation of carbamates in plants, the impact of carbamates on DOM composition, and the role of plant root secretion in the interaction between DOM and carbamates. Therefore, a hydroponic experiment instead of a soil experiment was designed to avoid the interference of microbial activity on the experimental results. The hydroponic experiments were designed based on our previous study [32]. The inorganic nutrient solution used for plant cultivation was based on the modified Hoagland formula. The exposure concentration of the three carbamates, that is, isoprocarb, carbofuran, and carbaryl, was set at 5 μg/mL, which is consistent with our previous work [32]. This was based on the maximum residue limits of carbofuran in cabbage (5 μg/g) as regulated by the National Food Safety Standard of China (GB 2763-2021) [33].

HA was adopted as a model of soil DOM. The concentration of HA in the solutions was set at 20 mg/L based on the concentrations found in plant-cultured soil reported in previous studies [4,34,35]. Chinese cabbages (*Brassica campestris* L. subsp. chinensis Makino (var. communis Tsen et Lee)) were grown from seeds in an inorganic nutrient solution for 16 days in a plant growth chamber under controlled cultivation conditions (temperature, day/night 25 °C/18 °C; light time, 12 h from 6 am to 6 pm; humidity, 65%). The plants were then randomly distributed into four miniature plant growth chambers, with each containing six plants with 4.5 L of nutrient solution. This ensured that the roots were in close contact with the nutrient solution throughout the 21-day tracking period. To compensate for moisture loss through evaporation and plant absorption, 100 mL of pure water was added daily to each chamber. Before the exposure experiment, nutrient solutions containing HA were mixed homogeneously in the dark. To explore the impact of carbamate exposure on DOM composition, four separate treatments were established as follows: (1) HA-supplemented nutrient solution without plant cultivation (HA-NoP); (2) HA-supplemented nutrient solution with carbamate exposure and without plant cultivation (HA/Carbamate-NoP); (3) cabbage plants cultured in HA-added nutrient solution (HA-Plants); and (4) cabbage plants cultured in HA-added nutrient solution with carbamate exposure (HA/Carbamate-Plants). A group of plants exposed to carbamates and cultured in an inorganic nutrient solution without HA addition (NoHA/Carbamate Plants) was used to investigate the influence of DOM on the uptake and transformation of carbamates by plants. Each group comprised three replicates.

To monitor the presence of carbamates and carbamate metabolites in plants, a previously proposed non-destructive in vivo analytical method was used [32]. The method involved the use of a biocompatible polyaminal solid-phase microextraction (SPME) fiber for in vivo sampling of living cabbage plants that were exposed to carbamates for specific durations (6 h, as well as 1, 2, 3, 4, 5, 7, 10, 14, and 21 d). During the in vivo monitoring period, six plants from the exposure group were randomly selected for in vivo sampling each day (*n* = 6). The sampling order for the carbamate-exposed plants was random. To minimize the potential for errors resulting from differences in physical and biological conditions such as temperature, light, and nutrient solution composition, these variables were kept constant throughout the 21-day experiment. The position of the plants within each growth chamber was changed daily. In addition, 100 mL of the nutrient solution from each plant growth chamber was collected for DOM analysis at experimental periods of 0, 7, 14, and 21 d. The DOM samples were filtered with 0.45 µm membrane and stored at −80 °C before extraction and analysis.

### 2.3. In Vivo Analysis of Carbamates and Carbamate Metabolites in Plants

In vivo SPME sampling was performed as described previously [32]. After each in vivo sampling process, the polyaminal fiber was directly introduced into the injection port of a gas chromatography–quadrupole time-of-flight mass spectrometer (GC-QTOF-MS) for instrumental analysis. Further details regarding the in vivo SPME sampling and GC-QTOF-MS analysis are provided in Text S1. The in vivo quantification procedures for carbamate and carbamate metabolites in plants are described in Text S2. Statistical analyses of the carbamate concentrations in the plants were performed using student’s *t*-test by the Statistical Product and Service Solutions (SPSS; version 26.0; Chicago, IL, USA).

### 2.4. Extraction and FT-ICR-MS Analysis of DOM

The DOM samples were acidified to pH 2 using hydrochloric acid (guaranteed reagent) prior to extraction. DOM extraction was performed using solid-phase extraction (SPE) procedures with PPL cartridges (Bond Elut PPL-200 mg 3 mL, Agilent, Santa Clara, CA, USA), which were frequently used in previous studies on DOM extraction for the FT-ICR-MS test [29,30]. Before extraction, the cartridges were activated with 20 mL methanol (HPLC grade) and rinsed with 20 mL ultrapure water, followed by 20 mL HCl-acidified ultrapure water (pH 2). Nutrient solution samples (100 mL) were then gravitationally passed through the PPL cartridges, followed by desalting with 30 mL of HCl-acidified ultrapure water (pH 2) and 20 mL of ultrapure water. The PPL cartridges were then dried with N_2_ gas and eluted with 3 mL of HPLC-grade methanol (HPLC grade). Prior to the FT-ICR-MS analysis, the eluates were diluted with an identical volume of ultrapure water.

A Bruker Solarix XR 7.0T FT-ICR-MS (Bruker, Berlin, Germany) was applied to the analysis of DOM samples under negative-ion mode ESI (−) at the Institute of Analysis, Guangdong Academy of Sciences, Guangzhou, China. The DOM eluates were injected into the electrospray ionization (ESI) source at a flow rate of 120 L/h. Other instrumental parameters were set as follows:4.0 kV capillary column entry voltage, −500 V capillary column end voltage, 4 M Word data size, 300 average scan; 100–1200 mass-to-charge ratio (*m*/*z*) range, and resolving power of ~260,000 at *m*/*z* 400. The ions accumulated in the impact chamber for 0.1 s and were transferred to the ICR chamber with a flight time of 0.7 ms. Suwannee River natural organic matter (SRNOM) was used as the standard sample for mass axis calibration of the instrument prior to DOM analysis. During FT-ICR-MS analysis, *m*/*z* 369.119106 was used as the internal standard for real-time calibration.

### 2.5. Formula Assignment and Data Analysis of DOM

All the FT-ICR MS spectra were externally calibrated with peak clusters of sodium trifluoroacetate and then internally calibrated with a known CHO mass series using the FTMSCalibrate algorithm [36] to achieve a mass accuracy within 1.0 ppm for the entire spectrum. The peaks found in both blank samples were removed. The formula assignment of FT-ICR-MS spectra was performed using the MATLAB-based FTMSCombine algorithm [37] with the following conditions: S/N ≥ 6; 0.3 ≤ H/C ≤ 2.25; 0 ≤ O/C ≤ 1.2; 4 ≤ 12C ≤ 50; 13C ≤ 2; 14N ≤ 5; 32S ≤ 3; 33S ≤ 1; double bond equivalent (DBE) ≥ 0 and *m*/*z* = 100–1000. To reduce the variability between replicates, only the molecular formulas observed in all three replicates for each treatment were analyzed further. This resulted in 2952–8814 molecular formulas per treatment after trimming. The relative signal intensity of each molecular formula in each replicate was calculated by normalizing the respective signal intensity of each formula with the sum of all signal intensities. The average values of the relative signal intensity in the three replicates were considered as the relative abundance of DOM compounds in a certain treatment. The molecular parameters, including the modification aromatic index (AImod), double bond equivalence (DBE), and nominal oxidation state of carbon (NOSC) were calculated (Text S3).

Based on the DOM molecular formula, the Van Krevelen digraph can be divided into nine categories [5,38], namely, saturated compounds (0 ≤ O/C ≤ 0.52, 1.5 ≤ H/C ≤ 2.2), aminosugars (0.52 ≤ O/C ≤ 0.71, 1.5 ≤ H/C ≤ 2.2, N > 0), carbohydrates (0.71 ≤ O/C ≤ 1.2, 1.5 ≤ H/C ≤ 2.4), lignin (0.1 ≤ O/C ≤ 0.67, 0.7 ≤ H/C ≤ 1.5), tannins (0.67 ≤ O/C ≤ 1.2, 0.5 ≤ H/C ≤ 1.5), unsaturated hydrocarbons (0 ≤ O/C ≤ 0.1, 0.7 ≤ H/C ≤ 1.5), condensed aromatics (0 ≤ O/C ≤ 0.67, 0.2 ≤ H/C ≤ 0.7), and other molecules.

## 3. Results and Discussion

### 3.1. Effects of DOM on Bioaccumulation and Biotransformation of Carbamates in Living Plants

To investigate the effects of DOM on the bioaccumulation of carbamates in plants, pesticide concentrations in living cabbage stems of the HA/Carbamate-Plant and NoHA/Carbamate-Plant groups were continuously tracked using a nondestructive in vivo analytical method as proposed in our previous work [32]. The method involved the use of a biocompatible polyaminal SPME fiber for the in vivo sampling of living cabbage plant stems (Figure S1). The quantification of carbamates and carbamate metabolites was based on pre-equilibrium SPME sampling of living plants calibrated using the sampling rate (*R*_*S*_) calibration method (Text S2, Figure S2).

**Figure 1:**
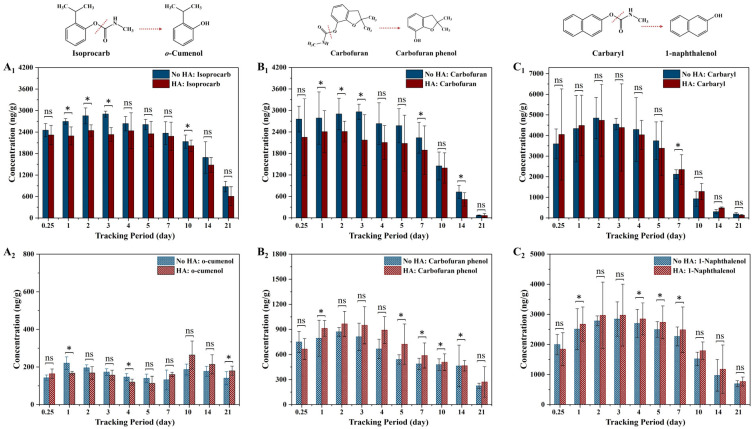
Molecular structures of carbamates and carbamate metabolites, and the concentrations of carbamates and carbamate metabolites in living cabbage plant stems of treatments with and without HA addition. (**A_1_**,**A_2_**) Concentrations of isoprocarb and o-cumenol, (**B_1_**,**B_2_**) concentrations of carbofuran and carbofuran phenol, and (**C_1_**,**C_2_**) concentrations of carbaryl and 1-naphthalenol. Significant difference between the two plant groups is represented as * *p* ≤ 0.05.

The temporal trends in the concentrations of carbamates and carbamate metabolites in the stems of living cabbage plants are shown in Figure 1. In plants exposed to carbamates without HA, that is, the NoHA/Carbamate-Plant group, the concentrations of three carbamates in the plant stems, isoprocarb, carbofuran, and carbaryl, increased rapidly within 24 h after carbamate treatment. The uptake of isoprocarb and carbofuran reached their highest mean concentrations on the third day of carbamate exposure at 2902 and 2962 ng/g, respectively. The peak mean carbaryl concentration reached 4846 ng/g on the second day of exposure. Then the concentrations of the three carbamates decreased over time.

With the presence of HA in the cultivation system, the highest mean concentrations of isoprocarb and carbofuran in plants cultured with HA were reached on the second days after exposure, respectively, which were different from those cultured without HA addition. The highest mean concentrations of isoprocarb and carbofuran in plants with HA addition were 2442 and 2411 ng/g, respectively, which were both lower than those in the plants in the NoHA/Carbamate-Plant group with reduced percentages of 16% and 19%. However, the presence of HA in the cultivation system did not significantly affect the uptake behavior of carbaryl in plants in that the highest concentrations of carbaryl and the time it took to reach the highest uptake amounts were both consistent in the two groups (*p* > 0.05). It could be concluded from the above results that the presence of DOM could reduce the highest bioconcentrations and decrease the time it took to reach the highest bioaccumulated amounts of isoprocarb and carbofuran in plants, while DOM did not show a significant effect on the uptake behavior of carbaryl.

The different effects of DOM on the bioaccumulation behaviors of the carbamate pesticides are thought to be related to their chemical structures. According to the structures of the three pesticides (Figure 1), in addition to the carbamate group, both isoprocarb and carbofuran have two methyl groups in their structures. The two methyl groups of isoprocarb are attached to the benzyl carbon and the methyl groups of carbofuran are attached to the oxygen-containing heterocyclic carbon. In contrast, carbaryl has only a biphenyl structure attached to a carbamate group. Isoprocarb and carbofuran have higher structural similarity and thus it was observed that their bioaccumulation behaviors were similar. In plants exposed to carbamates without HA, the uptake of isoprocarb and carbofuran reached their peak values on the third day of exposure with comparable bioconcentrations, while carbaryl possessed a much higher peak value and a shorter time to reach the peak concentration. Similarly, the effects of DOM on the bioaccumulation behaviors of isoprocarb and carbofuran were similar in that the presence of DOM could reduce the highest bioconcentrations and shorten the time to reach the highest bioaccumulated amounts of isoprocarb and carbofuran in plants, while it did not show significant effect on the uptake behavior of carbaryl.

In addition, the concentrations of three carbamate metabolites, namely, o-cumenol, carbofuran phenol, and 1-naphthalenol, were also measured on the first day of carbamate exposure (Figure 1), highlighting the rapid degradation of carbamate structures in living plants. The uptake, degradation, and elimination of carbaryl in the plants were the fastest among the three carbamates examined.

### 3.2. Carbamate-Induced Alterations of DOM Molecular Compositions

To investigate the effects of carbamate exposure on DOM composition, FT-ICR-MS was used for profiling of DOM in the different treatments. The detailed molecular parameters of the DOM properties are shown in Table 1. The formula numbers identified for DOM in the initial HA-supplemented nutrient solution with and without carbamate treatment (HA-NoP and HA/Carbamate-NoP groups) were 5643 and 5802, respectively. In the HA-NoP group, the number of identified DOM formulas increased from 5643 to 6802 on Day 7, 7096 on Day 14, and 7277 on Day 21, with increasing average *m*/*z* values. All the experimental groups were kept in plant growth chambers with intermittent circulatory pumps, regardless of whether the plants were cultivated. Therefore, owing to the promotion of HA dissolution by the circulatory pump in the plant growth chamber, the identified formula number of DOM gradually increased over the 21-day experimental period in the HA-NoP group. However, the identified formula number of DOM in the HA/Carbamate-NoP group first increased from 5802 to 6829 on Day 7, but then decreased to 2952 and 3701 on Days 14 and 21, respectively. The average *m*/*z* values decreased from 420 to 382 and 361, respectively. This indicates that carbamates might cause compositional sedimentation of DOM, thus resulting in the significant decrease of DOM numbers and average *m*/*z* values in the artificial hydroponic system.

In the plant cultivation treatments, the identified formula numbers of DOM in the HA-Plants and HA/Carbamate-Plant groups from Day 7 to Day 21 were 8308–8814 and 6048–8744, respectively. Compared to the HA-NoP group, plant cultivation significantly increased the number of DOM molecules in the HA-Plant group. In the HA/Carbamate-Plant group, the DOM number was 6048 in the first week after the exposure experiment. This was substantially lower than that of the plant cultivation group without carbamate exposure. However, in the second and third weeks, the number of DOM molecules in the two plant cultivation groups was comparable. From the perspective of changes in DOM numbers, plants induce an increase in DOM molecules through root secretion, whereas exposure to carbamates causes a decrease in DOM numbers. However, in the HA/Carbamate-Plant group, the decrease in DOM caused by carbamates was counteracted by plant cultivation. This implied that the plants gradually eliminated the impact of carbamate exposure on the DOM molecular numbers of the system through root secretion in the HA/Carbamate group. Additionally, as shown in Table 1, carbamate exposure led to a decrease in the average *m*/*z*, AImod and DBE values and an increase in the H/C value of DOM in the system, suggesting an increased lability of DOM caused by carbamates. Plant cultivation resulted in the maintenance of stable average *m*/*z* and AImod values of DOM, a slight increase in the H/C value, and a decrease in DBE. Similarly, in the HA/Carbamate-Plant group, the decrease in average *m*/*z* AImod and DBE values, as well as an increase in the H/C values of DOM caused by carbamate exposure, were partially counteracted by plant cultivation.

**Figure 2:**
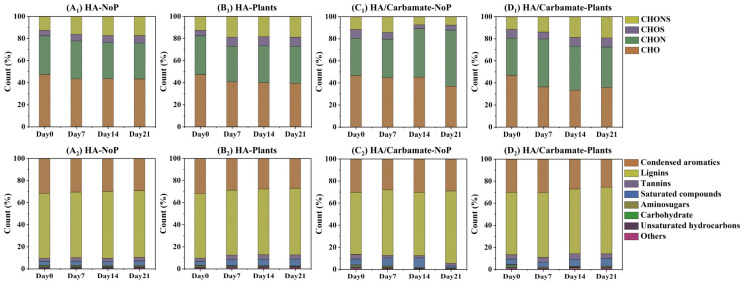
Relative amount of DOM molecules in different treatments according to (**A_1_**–**D_1_**) elemental compositions and (**A_2_**–**D_2_**) compound groups.

According to their elemental composition, all the DOM formulas fell into four major subcategories, that is, CHO (containing C, H, and O), CHON (containing C, H, O, and N), CHOS (containing C, H, O, and S), and CHONS (containing C, H, O, N, and S). As demonstrated in Figure 2, the CHO and CHON subcategories were the most abundant in all groups, accounting for 33–47% and 32–51%, respectively. In the treatments without carbamate exposure (HA-NoP and HA-Plant groups), the proportions of different DOM subcategories were altered in the first week and then remained stable for the next two weeks (Figure 2(A_1_,B_1_)). Nevertheless, the proportions of different DOM subcategories in the HA/Carbamate-NoP group varied continuously and substantially during the 21 days of the exposure experiment (Figure 2(C_1_)), indicating the compositional instability of DOM caused by carbamate treatment. In the HA/Carbamate-Plant group (Figure 2(D_1_)), the DOM composition changed during the first and second weeks and then remained stable during the third week. After 21 days of cultivation, the DOM compositions in the four elemental subcategories of the HA-Plant and HA/Carbamate-Plant groups showed a high degree of similarity (Figure S3A). Consistent with the results for the DOM molecular numbers, the DOM compositions of the different treatments also demonstrated that the plants could alleviate the impact of carbamate exposure on the DOM compositions of the system.

A van Krevelen diagram was used to provide a visual graphic display of the compound distribution, which showed differential changes in DOM molecular chemodiversity. Van Krevelen diagrams of the four treatments on Days 0, 7, 14, and 21 are shown in Figures S4–S7. According to the van Krevelen diagrams, saturated compounds, aminosugars, carbohydrates, unsaturated hydrocarbons, lignin, tannins, condensed aromatics, and other compounds were detected. Lignins were the most abundant molecules in the DOM originating from all treatments, ranging from 57% to 66%, followed by the condensed aromatics (26–32%) (Figure 2(A_2_–D_2_)). In the HA-NoP group, the proportions of the different DOM compounds remained stable during the 21 day experiment (Figure 2(A_2_)). In treatments with plant cultivation, that is, the HA-Plant group, the proportions of saturated compounds and tannins increased while the proportion of condensed aromatics decreased over time (Figure 2(B_2_)). In contrast, in the HA/Carbamate-NoP group, the proportions of most DOM compounds were reduced after three weeks of carbamate exposure, except for lignins (Figure 2(C_2_)), illustrating a reducing diversity of DOM compounds. After 21 days of cultivation, the proportions of different DOM compounds of the HA-Plant and HA/Carbamate-Plant groups also showed a high degree of similarity (Figure S3B). However, the changes in the proportions of different DOM compounds were altered more distinctly in the HA/carbamate-Plant group at the first and second weeks, as those of the non-contaminated treatment were mostly stable from Day 7 to Day 21. The results further indicated that the presence of plants enhanced the self-recovery of DOM in the system and alleviated the impact of carbamates on DOM composition.

### 3.3. Persistent, Removed, and Produced Compositions of DOM

**Figure 3:**
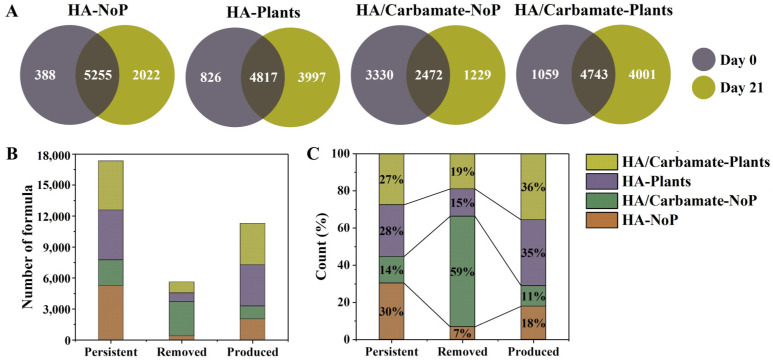
(**A**) Venn diagrams exhibiting the production, persistence, and removal of DOM molecules of different treatments between Day 0 and Day 21. The (**B**) molecular numbers and (**C**) relative amount of the persistent, removed, and produced DOM molecules in different treatments.

To elucidate the changes in DOM during the 21 day experiment, the removal, persistence, and production of DOM molecules in the different treatments were further analyzed. As illustrated in Figure 3, continuous substance exchange behavior of the DOM composition occurred in the different treatments. After 21 days of incubation, the number of DOM molecules in the HA-NoP group increased, and the number of molecules produced (2022) was substantially higher than that of the removed molecules (388). In contrast, the DOM numbers in the HA/Carbamate-Plant group decreased by 3330 with carbamate exposure. In contrast, plant cultivation could effectively promote the rate of substance exchange and generate more molecules, increasing the number and molecular diversity of DOM. A total of 3997 and 4001 molecules were produced in treatments with plant cultivation, respectively.

The changed molecules distributed in the four elemental subcategories and seven compound categories of DOM in different treatments were estimated (Figures S8 and S9). As shown in Figure S8, in the HA-NoP group, the DOM molecules produced were predominantly CHONS compounds, followed by CHON, CHO, and CHOS compounds. Based on the proportions of DOM molecules produced in the different subcategories, the proportion of CHOS and CHONS compounds produced were both the highest, reaching 48% of the total molecular number of CHOS. In contrast, the DOM numbers in the HA/Carbamate-NoP group decreased substantially. The removed proportions of the CHO, CHOS and CHONS compounds were considerably higher than the produced proportions, which was in contrast with those in the HA-NoP group. In the treatments with plant cultivation, that is, the HA Plants and HA/Carbamate-Plant groups, the rate of substance exchange and the amount of molecule generation were highly promoted. The removed and produced DOM numbers of the four subcategories in the plant cultivation treatments were comparable, regardless of carbamate exposure. The proportions of CHOS and CHONS compounds in these two treatments exceeded 50%, whereas the proportions of CHON and CHONS compounds exceeded 38% and 20%, respectively. As shown in Figure S8, the removed proportions of the CHOS and CHONS compounds were the highest among the four DOM subcategories in all treatments. However, the produced amounts of these two DOM subcategories in the HA-NoP group and the two treatments with plant cultivation were substantially higher than the removed ones. This led to a relatively high rate of increase in these compounds in these treatments, except for the HA/Carbamate-NoP group, where the produced amounts of CHON and CHONS compounds were considerably lower than those that were removed.

The changes of different compounds are illustrated in Figure S9. In the HA-NoP group, the proportion of tannins produced was the highest, followed by saturated compounds, aminosugars, lignin, condensed aromatics, and carbohydrates. However, in the HA/Carbamate-NoP group, the removed proportions of all the categories were relatively high among the different DOM compound categories, further demonstrating that exposure to carbamates substantially decreased the number and molecular diversity of DOM. Regarding the produced and removed proportions of different compound categories in the treatments with plant cultivation, the proportions of saturated compounds, aminosugars, lignin, tannins, and condensed aromatics were higher than those removed. Meanwhile, the proportions of carbohydrates and unsaturated hydrocarbons produced were lower than those removed.

### 3.4. Contribution of Root Secretion

The above results showed that plant cultivation induced higher turnover rates of DOM components and increased the amount and molecular diversity of DOM, thus strengthening the self-recovery function of the DOM system and alleviating the impacts of carbamates on DOM composition. To further elucidate the role of plant root secretion, the details of plant root secretion were investigated by comparing the DOM components of the control (the HA-NoP group) and two plant cultivated groups each week. As shown in the Venn diagram (Figures S10 and S11), plant culture increased the DOM components in the system through the generation of root secretions. However, plant cultivation also had an inhibitory effect, that is, an elimination effect, on some DOM components. These inhibited components may be absorbed or transformed by plants through root secretions or by reacting with root secretions.

In the HA-Plant group, after cultivation for 7, 14, and 21 d, the numbers of root exudates were 2362, 2669, and 2971, respectively. The numbers of inhibited DOM components were 856, 1173, and 1434, respectively, both of which gradually increased each week (Figure S10). By subtracting the number of root exudates and inhibited components, the net increase in DOM molecules in the plant cultivation group compared to the non-cultivation group was calculated to be 1506, 1496, and 1537 in the first, second, and third weeks, respectively. The increase in the amount of DOM substances contributed by plant cultivation was consistent each week. In the HA/Carbamate-Plant group (Figure S11), the number of root exudates was 1799, 3066, and 3096, and the number of inhibited DOM components was 2553, 1857, and 1629 at the first, second, and third weeks, respectively. The number of inhibited DOM components in the carbamate-treated plant cultivation group was substantially higher each week than that in the uncontaminated plant cultivation group, particularly in the first week. Moreover, the inhibited DOM molecules gradually decreased during the three-week experiment, in contrast to the uncontaminated plant cultivation group. The net increase in DOM molecules in the carbamate-treated plant cultivation group was also calculated to be −754, 1209, and 1467 in the first, second, and third weeks, respectively.

**Figure 4:**
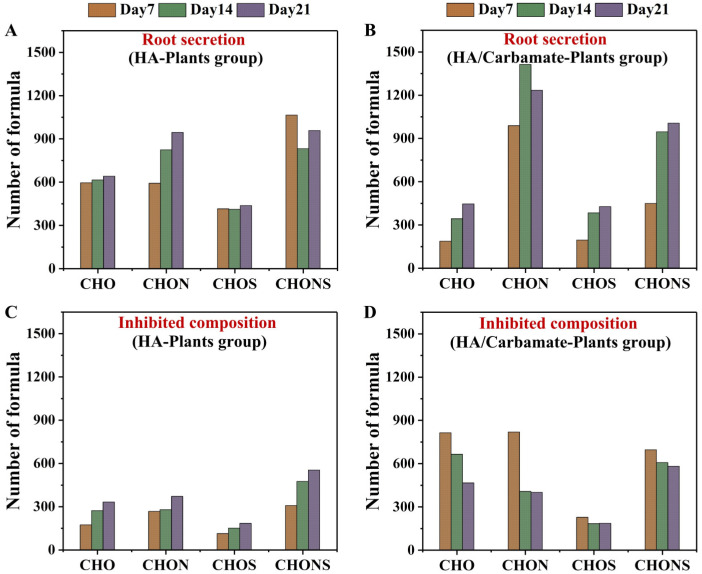
Difference in the root exudates and the inhibited DOM components of the HA-Plant and the HA/Carbamate-Plant groups during the 3-week experiment. The numbers of (**A**,**B**) the root exudates and (**C**,**D**) the inhibited DOM components in the four subcategories (CHO, CHON, CHONS, and CHOS) of the HA-Plant and the HA/Carbamate-Plant groups.

The number of different DOM subcategories among the root exudates and inhibited DOM components in the two plant cultivation groups each week is shown in Figure 4. In the HA-Plant group, the amount of root exudates and inhibited DOM components in the four subcategories, that is, CHO, CHON, CHONS, and CHOS, almost increased weekly. For all DOM subcategories each week, the number of root exudates was higher than that of the inhibited DOM components, resulting in a net increase in the number of DOM subcategories each week. In the HA/Carbamate-Plant group, the number of root exudates in different DOM subcategories also increased weekly, except for the CHON compounds. Nevertheless, the proportions of different DOM subcategories were quite different in the root exudates of the two plant cultivation groups. Meanwhile, the inhibited DOM components of different DOM subcategories in the carbamate-treated plant cultivation group showed a declining trend, in contrast to the uncontaminated group, and many more CHO compounds were inhibited in the carbamate-treated group.

By comparing the root exudates and inhibited DOM components of the two plant cultivation groups, we found that the carbamate treatment in the first week led to a substantial disturbance in the DOM composition of the plant cultivation system. This resulted in a distinctly greater number of inhibited DOM components and a lower number of root exudates in the carbamate-treated group. However, in the second and third weeks, the effect of carbamates was gradually alleviated by plant root secretions. Although exposure to carbamates increased the number of inhibited components in the system, the number of root exudates also increased. This resulted in a consistent net increase in the DOM molecules between the exposed and uncontaminated plant cultivation groups in the third week. It could be concluded that the turnover rate of the DOM components in the carbamate-treated plant cultivation group was higher than that in the uncontaminated group. This led to consistent proportions of different DOM subcategories and compound categories in the two plant cultivation groups in the third week (Figure S3), although carbamate treatment in the first week led to a significant disturbance in the DOM compositions. These results have further highlighted the conclusion that the presence of plants could enhance the self-cleaning function of the system and alleviate the impact of carbamates on DOM composition through root secretions.

## 4. Conclusions

The extensive and improper use of pesticides such as carbamates has caused severe pollution worldwide, posing a threat to human safety and environmental health [39,40]. Although the US, European and China partially restricted or banned some carbamate use in agriculture, carbamate consumption is still reported to be higher than that of several commonly used pesticides including pyrethroids, organophosphates, and organochlorines [24,25,26,32]. The potential risks and adverse impacts of carbamate pesticides are attracting considerable attention. In this study, the effect of DOM on the bioaccumulation and biotransformation of carbamates in plants was investigated. It was found that the presence of DOM could reduce the highest bioconcentrations and shorten the time it took to reach the highest bioaccumulated amounts of isoprocarb and carbofuran in plants, while DOM did not show significant effect on the uptake behavior of carbaryl. Meanwhile, the impact of carbamate exposure on DOM composition and the influence of plant root secretion on the interaction between DOM and carbamates were also studied. Carbamate pesticides were observed to reduce substantially the number and molecular diversity of DOM, while plant cultivation could effectively promote the rate of substance exchange and generate more molecules, increasing the number and molecular diversity of DOM. Owing to the complex compositions and diversified functions of DOM, changes in the molecular level of DOM induced by pesticides would inevitably alter its environmental functions and biogeochemical cycles. However, it was also found that plant root secretion could compensate for the DOM molecular changes induced by carbamate by inducing a higher turnover rate and producing more new components, demonstrating the role of plants in maintaining environmental DOM homeostasis and improving the resilience of DOM composition. Future research should further explore the influence of plant root secretion on the environmental behavior and harmful effects of different pesticides, thus pursuing green pollution control strategies.

## Figures and Tables

**Table 1:** An overview of the FT-ICR-MS average molecular parameters for DOM in different treatments.

Group	Time (d)	Formula Number	Measure *m*/*z*	H/C	O/C	N/C	S/C	AImod	DBE	NOSC
HA-NoP, HA-Plants	0	5643	413	0.816	0.412	0.023	0.007	0.551	13.7	0.076
HA-NoP	7	6802	434	0.812	0.427	0.025	0.008	0.547	14.2	0.123
	14	7096	444	0.808	0.428	0.024	0.008	0.546	14.5	0.126
	21	7277	444	0.813	0.427	0.024	0.008	0.543	14.4	0.120
HA-Plants	7	8308	436	0.829	0.449	0.026	0.011	0.529	13.8	0.161
	14	8592	430	0.862	0.447	0.027	0.011	0.510	13.3	0.132
	21	8814	426	0.881	0.442	0.027	0.011	0.501	13.0	0.102
HA/Carbamate-NoP, HA/Carbamate-Plants	0	5802	420	0.897	0.401	0.022	0.012	0.507	12.5	−0.0243
HA/Carbamate-NoP	7	6829	423	1.109	0.404	0.031	0.018	0.404	10.9	−0.179
	14	2952	382	1.160	0.441	0.143	0.027	0.412	9.13	0.199
	21	3701	361	0.946	0.472	0.127	0.007	0.485	10.37	0.390
HA/Carbamate-Plants	7	6048	420	0.858	0.412	0.024	0.011	0.524	13.4	0.045
	14	8305	439	0.883	0.455	0.040	0.012	0.492	13.2	0.164
	21	8744	428	0.893	0.450	0.033	0.012	0.486	12.9	0.125

## Data Availability

Data are contained within the article or Supplementary Materials. Further inquiries can be directed to the corresponding authors.

## Supplementary Materials

The following supporting information can be downloaded at: https://www.mdpi.com/article/10.3390/toxics12090654/s1, Text S1: In vivo SPME sampling and instrument analysis [32]; Text S2: In vivo quantification procedures; Text S3: Calculation of DOM parameters [41,42,43]; Figure S1: Image of polyaminal fibers, in vivo sampling and instrumental analysis; Figure S2: Quantitative curves; Figure S3: Relative amount of DOM molecules in different treatments; Figures S4–S7: van Krevelen diagrams; Figures S8 and S9: The molecular numbers and relative amount of the persistent, removed, and produced DOM molecules; Figures S10 and S11: Illustration of the plant root secretion and inhibited components.

## References

[1] Phillips A.A., White M.E., Seidel M., Wu F., Pavia F.F., Kemeny P.C., Ma A.C., Aluwihare L.I., Dittmar T., Sessions A.L. (2022). Novel sulfur isotope analyses constrain sulfurized porewaterfluxes as a minor component of Marine Dissolved Organic Matter. Proc. Natl. Acad. Sci. USA.

[2] Osterholz H., Turner S., Alakangas L.J., Tullborg E.L., Dittmar T., Kalinowski B.E., Dopson M. (2022). Terrigenous Dissolved Organic Matter Persists in the Energy-Limited Deep Groundwaters of the Fennoscandian Shield. Nat. Commun..

[3] Witzgall K., Vidal A., Schubert D.I., Höschen C., Schweizer S.A., Buegger F., Pouteau V., Chenu C., Mueller C.W. (2021). Particulate Organic Matter as a Functional Soil Component for Persistent Soil Organic Carbon. Nat. Commun..

[4] Guo J., Zhou J., Liu S., Shen L., Liang X., Wang T., Zhu L. (2022). Underlying Mechanisms for Low-Molecular-Weight Dissolved Organic Matter to Promote Translocation and Transformation of Chlorinated Polyfluoroalkyl Ether Sulfonate in Wheat. Environ. Sci. Technol..

[5] Sun Y., Li X., Li X., Wang J. (2022). Deciphering the Fingerprint of Dissolved Organic Matter in the Soil Amended with Biodegradable and Conventional Microplastics Based on Optical and Molecular Signatures. Environ. Sci. Technol..

[6] Kalčíková G., Zupančič M., Jemec A., Žgajnar Gotvajn A. (2016). The Impact of Humic Acid on Chromium Phytoextraction by Aquatic Macrophyte Lemna Minor. Chemosphere.

[7] Ding Y., Bai X., Ye Z., Gong D., Cao J., Hua Z. (2019). Humic Acid Regulation of the Environmental Behavior and Phytotoxicity of Silver Nanoparticles to: Lemna Minor. Environ. Sci. Nano.

[8] Liang Y., He J., Zhang S., Xia Q. (2021). Effects of Exogenous Organic Matter on the Migration of Organic Contaminants with Different Polarities in Soil. Int. J. Environ. Res..

[9] Zhu S., Mo Y., Luo W., Xiao Z., Jin C., Qiu R. (2023). Dissolved Organic Matter Regulates Aggregation and Deposition of Chromium (Hydr)Oxide Colloids: Molecular-Scale Investigation Using ESI-FT-ICR-MS. Environ. Sci. Nano.

[10] Fadare O.O., Wan B., Liu K., Yang Y., Zhao L., Guo L.H. (2020). Eco-Corona vs Protein Corona: Effects of Humic Substances on Corona Formation and Nanoplastic Particle Toxicity in Daphnia Magna. Environ. Sci. Technol..

[11] Gao Y., Zhu J., He A. (2022). Effect of Dissolved Organic Matter on the Bioavailability and Toxicity of Cadmium in Zebrafish Larvae: Determination Based on Toxicokinetic–Toxicodynamic Processes. Water Res..

[12] Ren Z., Zhang H., Wang Y., Lu L., Ren D., Wang J. (2021). Multiple Roles of Dissolved Organic Matter Released from Decomposing Rice Straw at Different Times in Organic Pollutant Photodegradation. J. Hazard. Mater..

[13] He W., Bai Z.L., Li Y.L., Kong X.Z., Liu W.X., Yang C., Yang B., Xu F.L. (2016). Advances in Environmental Behaviors and Effects of Dissolved Organic Matter in Aquatic Ecosystems. Sci. China Earth Sci..

[14] Sheridan E.A., Fonvielle J.A., Cottingham S., Zhang Y., Dittmar T., Aldridge D.C., Tanentzap A.J. (2022). Plastic Pollution Fosters More Microbial Growth in Lakes than Natural Organic Matter. Nat. Commun..

[15] Chen W., Teng C.Y., Qian C., Yu H.Q. (2019). Characterizing Properties and Environmental Behaviors of Dissolved Organic Matter Using Two-Dimensional Correlation Spectroscopic Analysis. Environ. Sci. Technol..

[16] Sun Y., Ji J., Tao J., Yang Y., Wu D., Han L., Li S., Wang J. (2023). Current Advances in Interactions between Microplastics and Dissolved Organic Matters in Aquatic and Terrestrial Ecosystems. Trends Anal. Chem..

[17] FAO Pesticides Use (FAOSTAT). https://www.fao.org/faostat/en/#data/RP.

[18] Maggi F., Tang F.H.M., Tubiello F.N. (2023). Agricultural Pesticide Land Budget and River Discharge to Oceans. Nature.

[19] Panico S.C., van Gestel C.A.M., Verweij R.A., Rault M., Bertrand C., Menacho Barriga C.A., Coeurdassier M., Fritsch C., Gimbert F., Pelosi C. (2022). Field Mixtures of Currently Used Pesticides in Agricultural Soil Pose a Risk to Soil Invertebrates. Environ. Pollut..

[20] Cao Y., Ibáñez Navarro A., Perrella L., Cedergreen N. (2021). Can Organophosphates and Carbamates Cause Synergisms by Inhibiting Esterases Responsible for Biotransformation of Pyrethroids?. Environ. Sci. Technol..

[21] Jeong D.H., Jung D.W., Jang C.H., Kim U.J., Park Y., Park Y., Lee H.S. (2023). Chlorpropham, a Carbamate Ester Herbicide, Has an Endocrine-Disrupting Potential by Inhibiting the Homodimerization of Human Androgen Receptor. Environ. Pollut..

[22] Thirumalraj B., Krishnapandi A., Chen S.M., MSP S., Choe H. (2020). Rational Design and Interlayer Effect of Dysprosium-Stannate Nanoplatelets Incorporated Graphene Oxide: A Versatile and Competent Electrocatalyst for Toxic Carbamate Pesticide Detection in Vegetables. ACS Sustain. Chem. Eng..

[23] Gupta J., Rathour R., Singh R., Thakur I.S. (2019). Production and Characterization of Extracellular Polymeric Substances (EPS) Generated by a Carbofuran Degrading Strain Cupriavidus Sp. ISTL7. Bioresour. Technol..

[24] Zhang J., Guo J., Wu C., Qi X., Jiang S., Lv S., Lu D., Liang W., Chang X., Zhang Y. (2022). Carbamate Pesticides Exposure and Delayed Physical Development at the Age of Seven: Evidence from the SMBCS Study. Environ. Int..

[25] Zhang J., Guo J., Wu C., Qi X., Jiang S., Zhou T., Xiao H., Li W., Lu D., Feng C. (2020). Early-life carbamate exposure and intelligence quotient of seven-year-old children. Environ. Int..

[26] (2017). Ministry of Agriculture and Rural Affairs of the People’s Republic of China. List of Restricted Pesticides. http://www.moa.gov.cn/govpublic/ZZYGLS/201709/t20170911_5810706.htm.

[27] Liu L., Tsyusko O.V., Unrine J.M., Liu S., Liu Y., Guo L., Wei G., Chen C. (2023). Pristine and Sulfidized Zinc Oxide Nanoparticles Promote the Release and Decomposition of Organic Carbon in the Legume Rhizosphere. Environ. Sci. Technol..

[28] Zhou Z., Fu Q.L., Fujii M., Waite T.D. (2023). Complementary Elucidation of the Molecular Characteristics of Groundwater Dissolved Organic Matter Using Ultrahigh-Resolution Mass Spectrometry Coupled with Negative- and Positive-Ion Electrospray Ionization. Environ. Sci. Technol..

[29] Zhao P., Du Z., Fu Q., Ai J., Hu A., Wang D., Zhang W. (2023). Molecular Composition and Chemodiversity of Dissolved Organic Matter in Wastewater Sludge via Fourier Transform Ion Cyclotron Resonance Mass Spectrometry: Effects of Extraction Methods and Electrospray Ionization Modes. Water. Res..

[30] Leyva D., Jaffe R., Fernandez-Lima F. (2020). Structural Characterization of Dissolved Organic Matter at the Chemical Formula Level Using TIMS-FT-ICR MS/MS. Anal. Chem..

[31] Zark M., Dittmar T. (2018). Universal Molecular Structures in Natural Dissolved Organic Matter. Nat. Commun..

[32] Liu S., Huang Y., Liu J., Chen C., Ouyang G. (2021). In Vivo Contaminant Monitoring and Metabolomic Profiling in Plants Exposed to Carbamates via a Novel Microextraction Fiber. Environ. Sci. Technol..

[33] (2021). National Standard for Food Safety: Maximum residue Limit of Pesticides in Food.

[34] Wang Y.F., Zhang X.Y., Zhang X., Meng Q.J., Gao F.J., Zhang Y. (2017). Characterization of Spectral Responses of Dissolved Organic Matter (DOM) for Atrazine Binding During the Sorption Process onto Black Soil. Chemosphere.

[35] Yu H., Huang G.H., An C.J., Wei J. (2011). Combined Effects of DOM Extracted from Site Soil/compost and Biosurfactant on the Sorption and Desorption of PAHs in a Soil-water System. J. Hazard. Mater..

[36] Fu Q.L., Fujii M., Kwon E. (2022). Development of an Internal Calibration Algorithm for Ultrahigh-Resolution Mass Spectra of Dissolved Organic Matter. Anal. Chem..

[37] Fu Q.L., Fujii M., Ma R. (2023). Development of a Gaussian-Based Alignment Algorithm for the Ultrahigh-Resolution Mass Spectra of Dissolved Organic Matter. Anal. Chem..

[38] Wu S., You F., Boughton B., Liu Y., Nguyen T.A., Wykes J., Southam G., Robertson L.M., Chan T.-S., Lu Y.-R. (2021). Chemodiversity of dissolved organic matter and its molecular changes driven by rhizosphere activities in Fe Ore tailings undergoing Eco-Engineered pedogenesis. Environ. Sci. Technol..

[39] Schulz R., Bub S., Petschick L.L., Stehle S., Wolfram J. (2021). Applied Pesticide Toxicity Shifts toward Plants and Invertebrates, Even in GM Crops. Science.

[40] Rumschlag S.L., Mahon M.B., Hoverman J.T., Raffel T.R., Carrick H.J., Hudson P.J., Rohr J.R. (2020). Consistent Effects of Pesticides on Community Structure and Ecosystem Function in Freshwater Systems. Nat. Commun..

[41] Koch B.P., Dittmar T., Witt M., Kattner G. (2007). Fundamentals of molecular formula assignment to ultrahigh resolution mass data of natural organic matter. Anal. Chem..

[42] Stenson A.C., Landing W.M., Marshall A.G., Cooper W.T. (2002). Ionization and fragmentation of humic substances in electrospray ionization Fourier transform-ion cyclotron resonance mass spectrometry. Anal. Chem..

[43] Riedel T., Biester H., Dittmar T. (2012). Molecular fractionation of dissolved organic matter with metal salts. Environ. Sci. Technol..

