# Supplementary material for: Plant Root Secretion Alleviates Carbamate-Induced Molecular Alterations of Dissolved Organic Matter
Source: Toxics. 2024 Sep 5;12(9):654. doi: 10.3390/toxics12090654 (PMC11435816; doi:10.3390/toxics12090654)
Supplement: Supplementary file 1 [file toxics-12-00654-s001.zip › toxics-3159519-supplementary.pdf]

*Supplementary Material*

# Plant Root Secretion Alleviates Carbamate-Induced Molecular Alterations of Dissolved Organic Matter

Zihan Niu [1], Chao Chen [2], Qijun Ruan [2], Yingming Duan [3], Shuqin Liu [1,2,*], Da Chen [1]

[1] *Guangdong Key Laboratory of Environmental Pollution and Health, School of Environment and Climate, Jinan University, Guangzhou 511443, China*

[2] *Guangdong Provincial Key Laboratory of Chemical Measurement and Emergency Test Technology, Institute of Analysis, Guangdong Academy of Sciences (China National Analytical Center Guangzhou), Guangzhou 510070, China*

[3] *College of Resources and Environmental Engineering, Guizhou University, Guiyang 550025, China*

* Corresponding author. Tel. & Fax: +86-2037336651

E-mail: liushuqin@jnu.edu.cn

**Text S1. In Vivo SPME Sampling and Instrument Analysis.**

The in vivo solid-phase microextraction (SPME) method [1] involved the use of a biocompatible polyaminal SPME fiber (**Figure S1A**) for in vivo sampling of living cabbage plants. Before in vivo SPME sampling, the SPME fiber was conditioned under a nitrogen atmosphere for 30 min at 250 °C to avoid any carry-over of the fiber coating. The sampling design comprised inserting the fiber coating into the plant stem tissue from a direction parallel to the stem (**Figure S1B**). The polyaminal fiber was then exposed to the tissue for 20 min at a sampling depth of 1.5 cm. After each in vivo sampling process, the fiber was directly introduced to the injection port of a gas chromatographic quadrupole time-of-flight mass spectrometer (GC-QTOF-MS) for 5 min (**Fig.S1C**), followed by an additional condition at 250 °C for 5 min.

GC-QTOF-MS analyses were conducted using an Agilent 7890B GC instrument coupled with a 7250 QTOF-MS instrument (Agilent Technologies, Santa Clara, CA). The GC column was an Agilent HP-5 MS column (30 m long, 250 µm i.d., with a 0.25 µm-thick 5% phenyl–95% dimethylpolysiloxane film). The SPME fiber was introduced into the splitless injector system of GC-QTOF-MS after in vivo sampling. The MS parameters were set as follows: electron impact ionization, 70 eV; transfer line temperature, 280 °C; ion source temperature, 200 °C and MS quadrupole temperature, 150 °C. The MS system was routinely set in a scan mode. The carrier gas was helium with constant flow rate of 1.7 mL $min^{-1}$. The oven temperature program started at 50 °C, increased at 20 °C $min^{-1}$ to 100 °C , then increased at 10 °C $min^{-1}$ to 220 °C, and then increased at 20 °C $min^{-1}$ to 280 °C; the total analysis time is 17.5 min.

**Text S2. In Vivo Quantification of Carbamates and Carbamate Metabolites in Plants.**

Pre-equilibrium SPME sampling of living plants was calibrated with the sampling rate ($R_s$) calibration method, as described in our previous work [1]. Briefly, in vivo concentrations of carbamates and carbamate metabolites in the living plant stems were calculated according to the following equation:

$$C_s = \frac{n}{R_s t} \quad (1)$$

in which $n$ is the extracted amount of fiber coating, $C_s$ is the analyte concentration in stem tissues determined via solvent extraction methods, and $t$ is the in vivo sampling duration.

The $R_s$ values of three carbamates and three carbamate metabolites in cabbage plant stems were detected in our previous work [1], which are 4.32, 1.53, 0.35, 1.80, 1.03, and 0.89 mg/min for *o*-cumenol, carbofuran phenol, 1-naphthalenol, isoprocarb, carbofuran, and carbaryl, respectively. The extracted amount of each target analyte in the fiber coating $n$ can be calculated by the quantitative curves of the standard solutions of carbamates and carbamate metabolites (**Figure S2**), while the sampling duration $t$ is 20 min. Then, the in vivo concentrations of carbamates and carbamate metabolites $C_s$ can be calculated by **Equation 1**.

**Text S3. Calculation of DOM Parameters.**

Aromaticity index (AI) was calculated from the formulas to estimate the fraction of aromatic and condensed aromatic groups (**Equation 2**) [2]. Double bond equivalence (DBE) was used to measure the number of double bonds and rings in a molecule (**Equation 3**) [3]. The nominal oxidation state of carbon (NOSC) representing the average oxidation state of all carbons per formula independent of the chemical structure was calculated (**Equation 4**) [4]:

$$\mathrm{DBE} = 1 + \frac{1}{2}(2n\mathrm{C} - n\mathrm{H} + n\mathrm{N}) \tag{2}$$

$$\mathrm{AI_{mod}} = \frac{1 + n\mathrm{C} - 0.5n\mathrm{O} - n\mathrm{S} - 0.5n\mathrm{H}}{n\mathrm{C} - 0.5n\mathrm{O} - n\mathrm{S} - n\mathrm{N}} \tag{3}$$

$$\mathrm{NOSC} = 4 - \frac{4n\mathrm{C} + n\mathrm{H} - 3n\mathrm{N} - 2n\mathrm{O} - 2n\mathrm{S}}{n\mathrm{C}} \tag{4}$$

where $n$C, $n$H, $nO$, $n$N, and $n$S, refer to the stoichiometric number of C, H, O, N, and S atoms per formula, respectively.

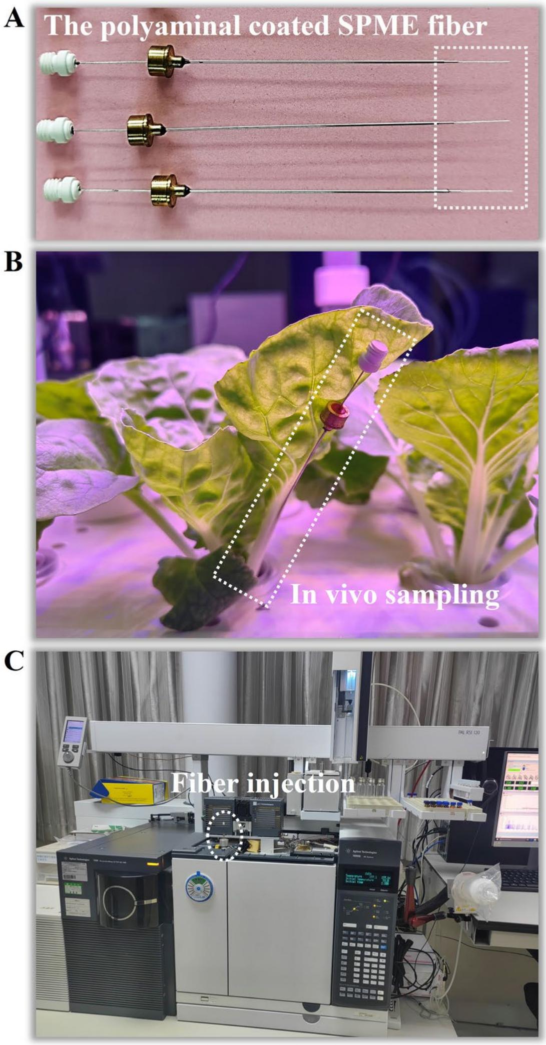


**Figure S1.** (A) Image of polyaminal fibers assembled into the SPME fiber assembly, (B) in vivo SPME sampling of the living cabbage plant stem, (C) analysis of carbamates and carbamate metabolites by introducing the sampled fiber into the GC-QTOF injection port.

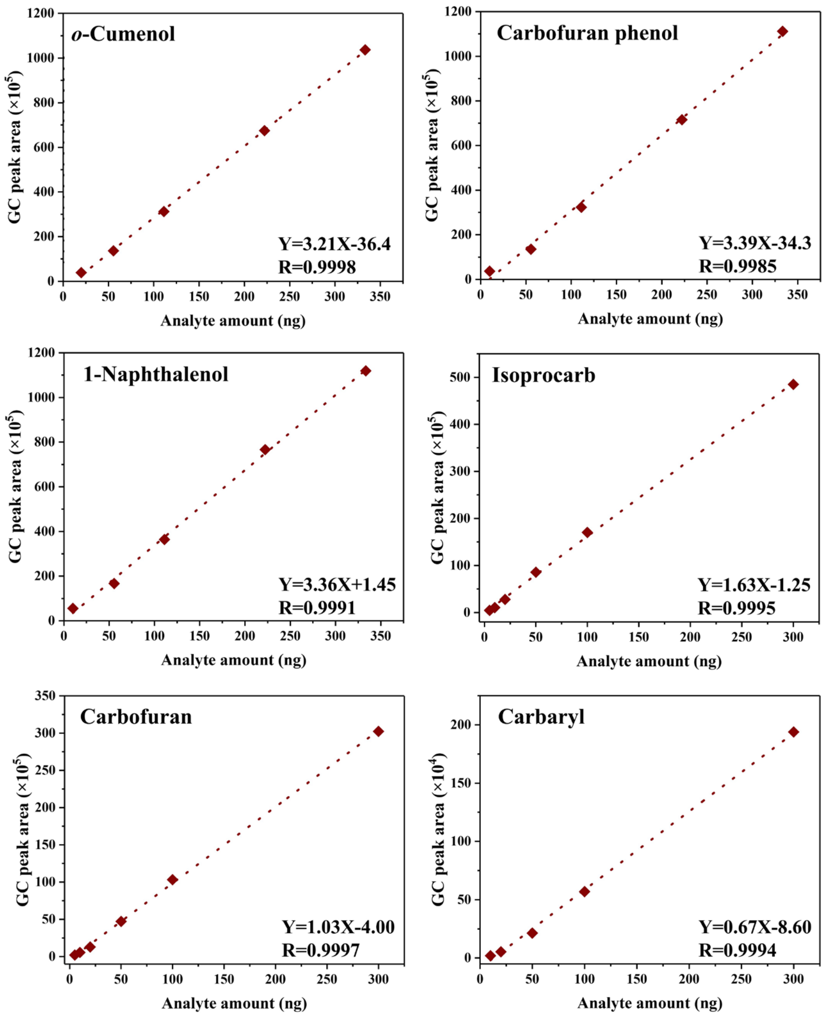


**Figure S2.** Quantitative curves of the standard solutions of carbamates and carbamate metabolites[1].

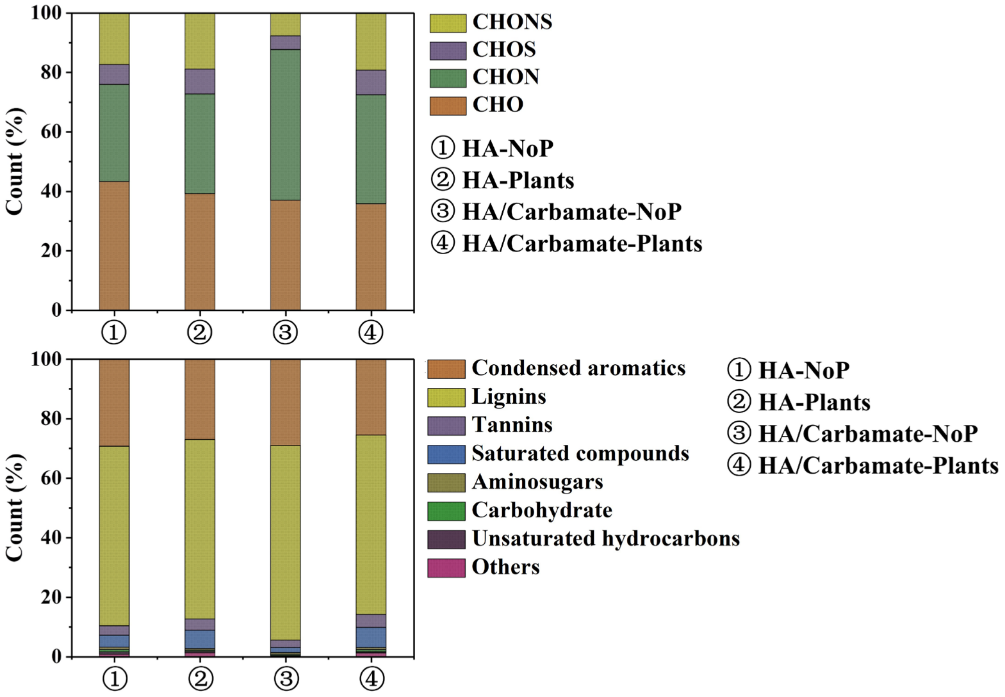


**Figure S3.** Relative amount of DOM molecules in different treatments on Day 21 according to (A) elemental compositions and (B) compound groups.

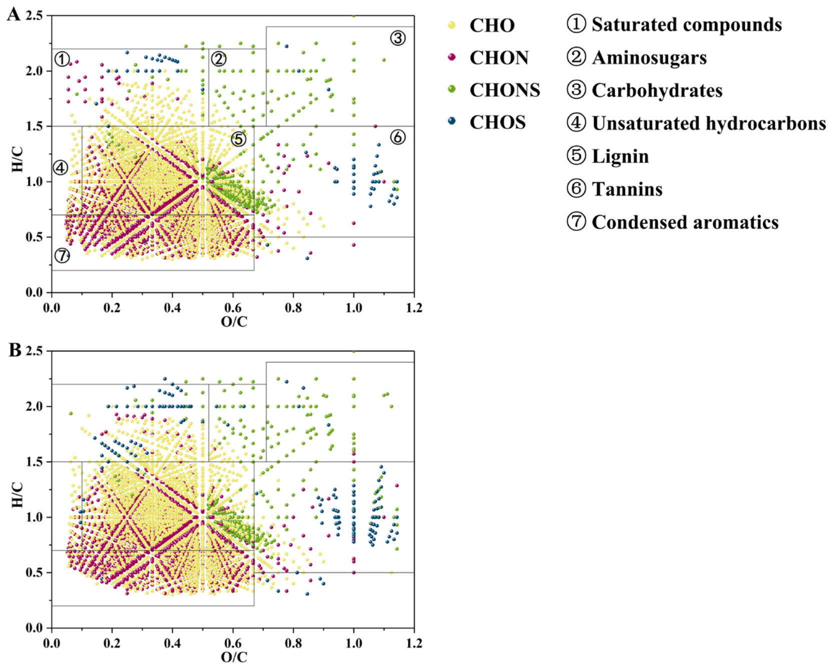


**Figure S4.** van Krevelen diagrams of CHO, CHON, CHONS, CHON on Day 0 of different treatments: (A) the HA-NoP group and the HA-Plants group, (B) the HA/Carbamate-NoP group and the HA/Carbamate-Plants group.

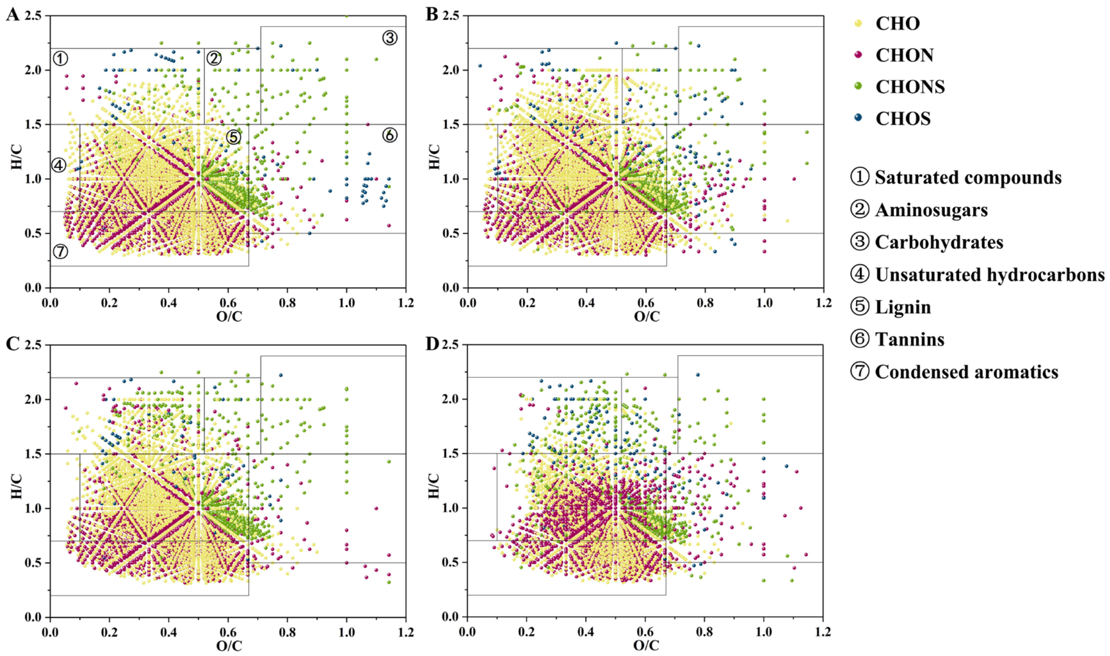


**Figure S5.** van Krevelen diagrams of CHO, CHON, CHONS, CHON on Day 7 of different treatments: (A) the HA-NoP group, (B) the HA-Plants group, (C) the HA/Carbamate-NoP group, and (D) the HA/Carbamate-Plants group.

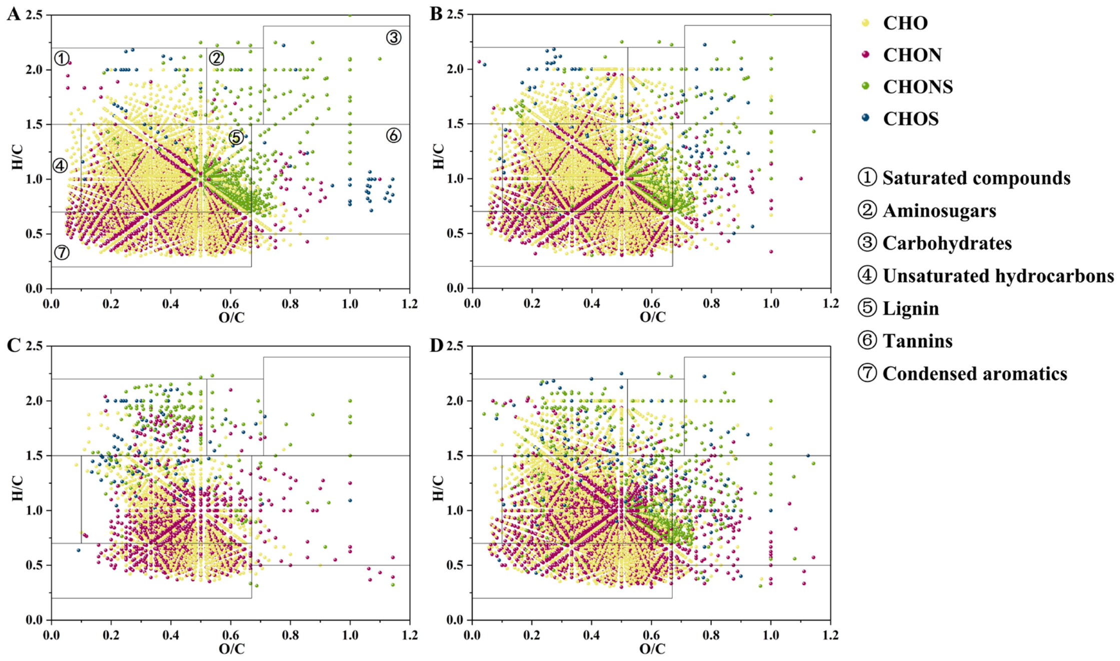


**Figure S6.** van Krevelen diagrams of CHO, CHON, CHONS, CHON on Day 14 of different treatments: (A) the HA-NoP group, (B) the HA-Plants group, (C) the HA/Carbamate-NoP group, and (D) the HA/Carbamate-Plants group.

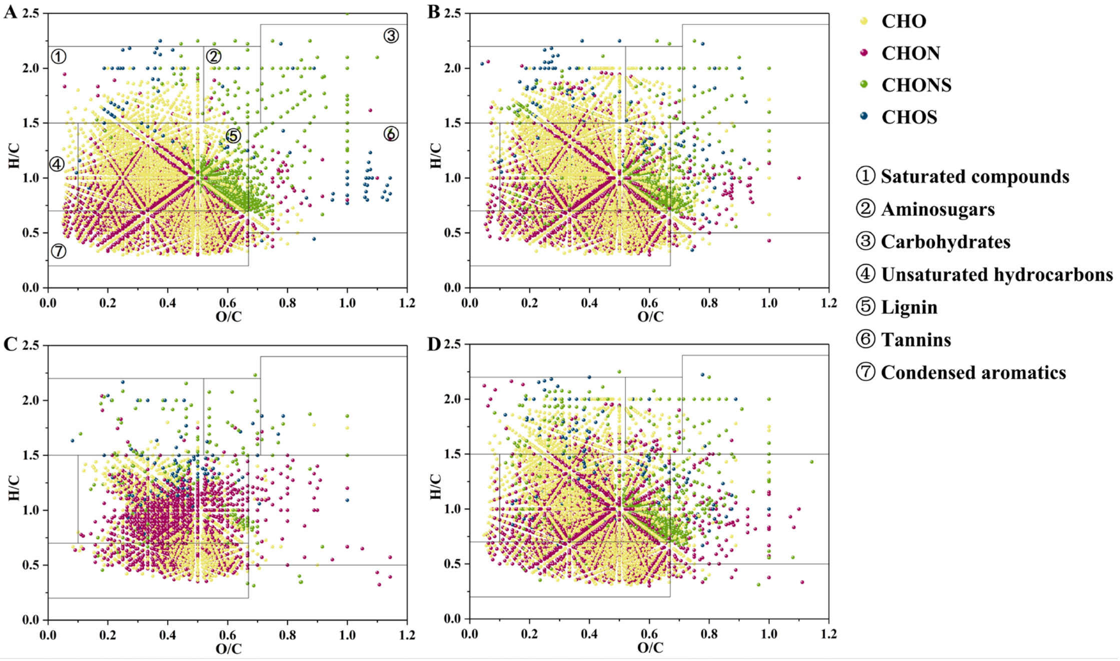


**Figure S7.** van Krevelen diagrams of CHO, CHON, CHONS, CHON on Day 21 of different treatments: (A) the HA-NoP group, (B) the HA-Plants group, (C) the HA/Carbamate-NoP group, and (D) the HA/Carbamate-Plants group.

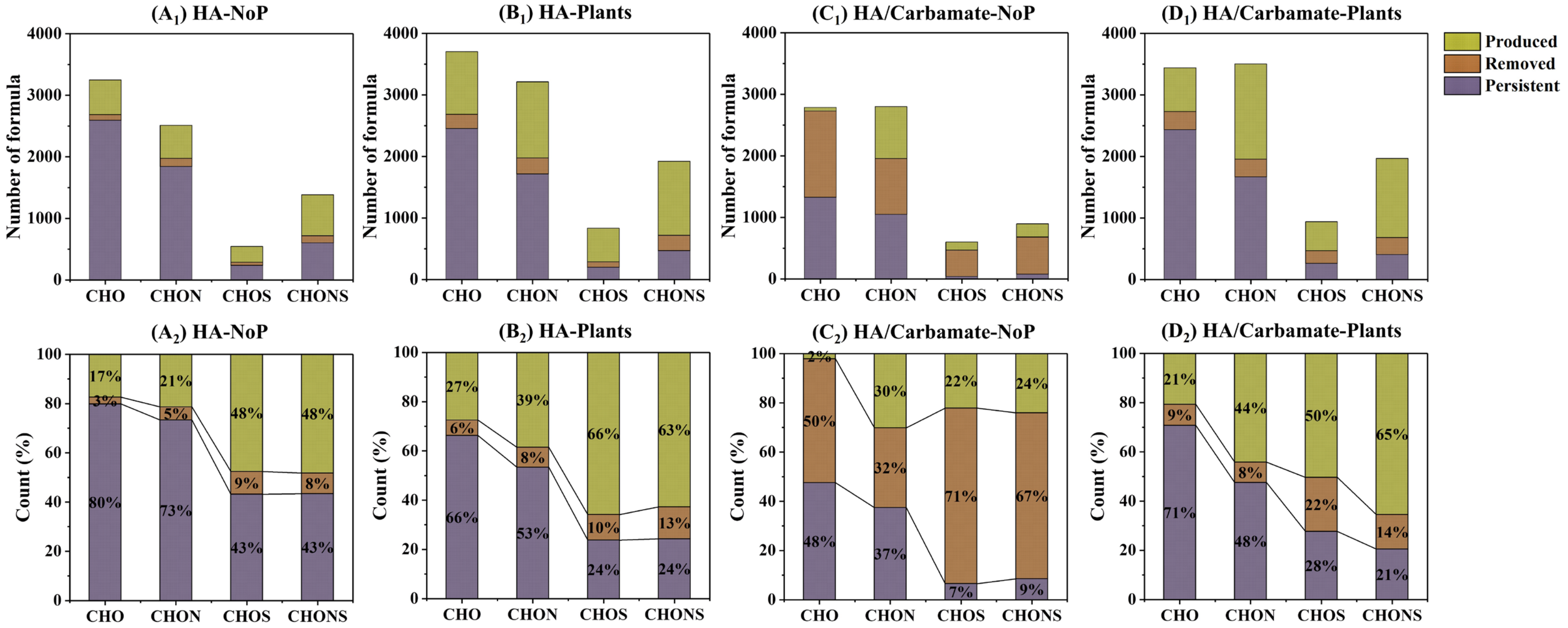


**Figure S8.** The ($A_1$-$D_1$) molecular numbers and ($A_2$-$D_2$) relative amount of the persistent, removed, and produced DOM molecules in the four subcategories (CHO, CHON, CHONS, and CHOS) of different treatments.

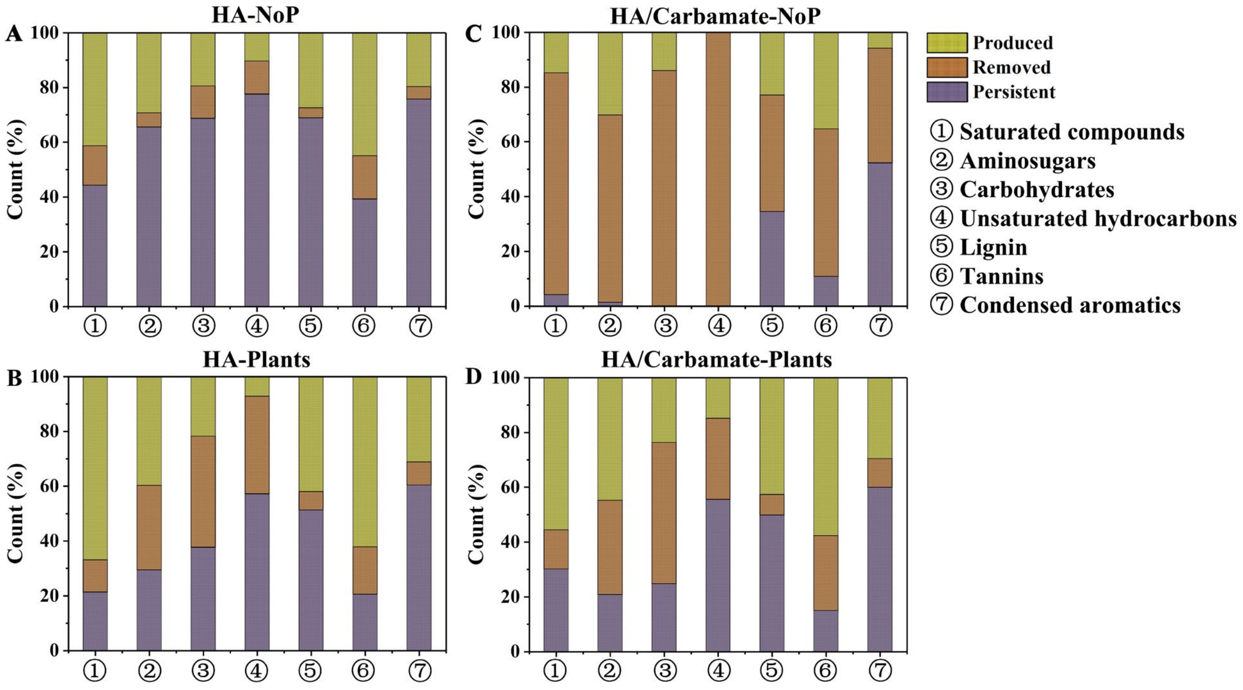


**Figure S9.** The relative amount of the persistent, removed, and produced DOM molecules in the seven compound groups of different treatments. (A) the HA-NoP group, (B) the HA-Plants group, (C) the HA/Carbamate-NoP group, and (D) the HA/Carbamate-Plants group.

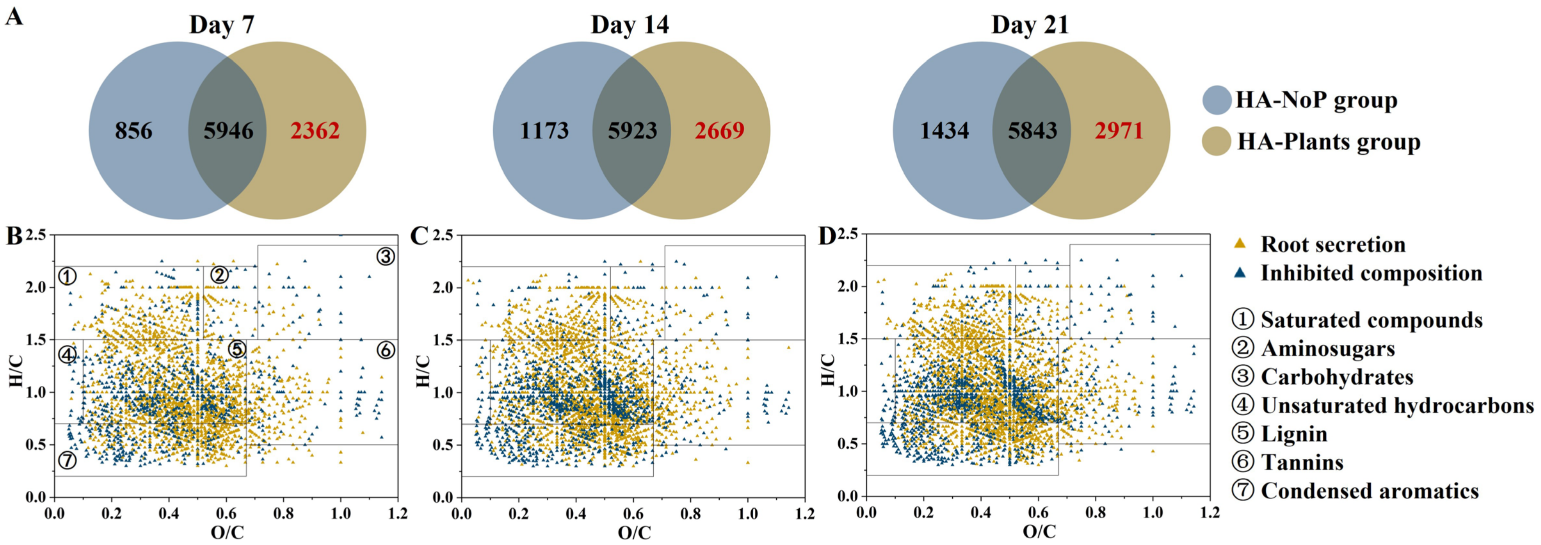


**Figure S10.** (A) Venn diagrams exhibiting the plant root secretion and inhibited components of DOM in the HA-Plants group by comparing the DOM components between the control group (the HA-NoP group) and the plant-cultivated group (the HA-Plants group) each week. van Krevelen diagrams exhibit the plant root secretion and inhibited components of DOM in the HA-Plants group on (A) Day 7, (B) Day 14, and (C) Day 21.

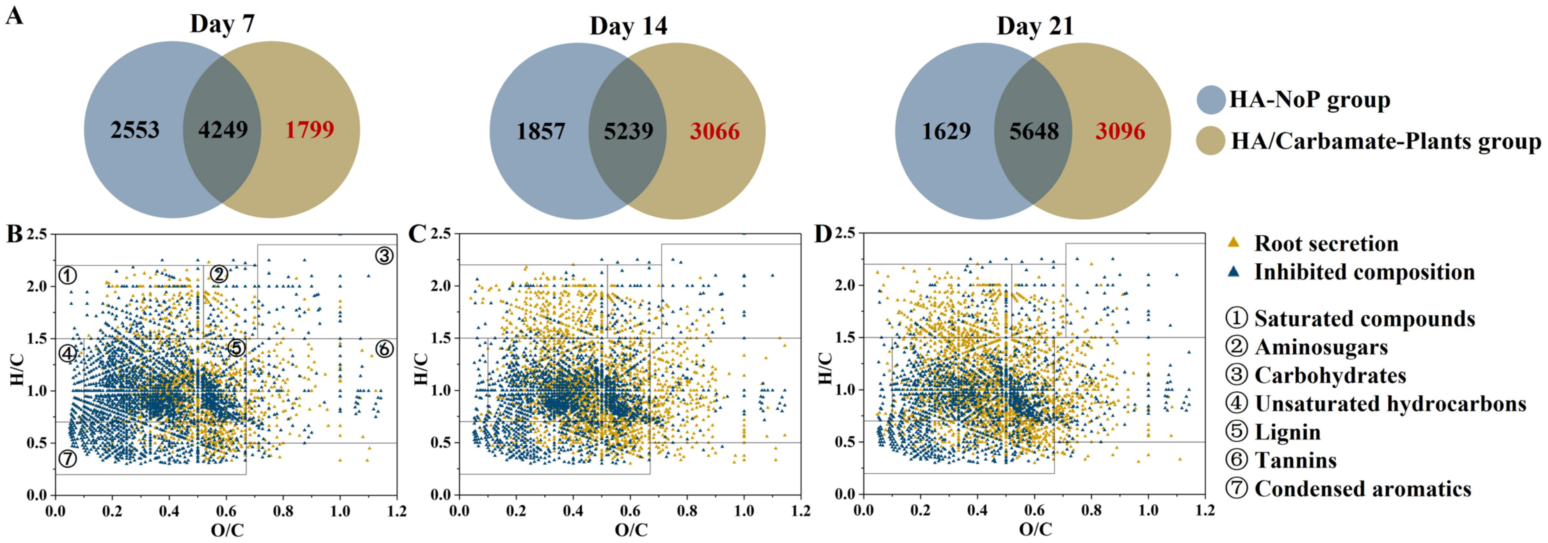


**Figure S11.** (A) Venn diagrams exhibiting the plant root secretion and inhibited components of DOM in the HA/Carbamate-Plants group by comparing the DOM components between the control group (the HA-NoP group) and the plant-cultivated group (the HA/Carbamate-Plants group) each week. van Krevelen diagrams exhibit the plant root secretion and inhibited components of DOM in the HA-Plants group on (A) Day 7, (B) Day 14, and (C) Day 21.

**References:**

[1] Liu, S.; Huang, Y.; Liu, J.; Chen, C.; Ouyang, G., 2021. In vivo contaminant monitoring and metabolomic profiling in plants exposed to carbamates via a novel microextraction fiber. Environ. Sci. Technol. 55 (18), 12449-12458.

[2] Koch, B. P.; Dittmar, T.; Witt, M.; Kattner, G., 2007. Fundamentals of molecular formula assignment to ultrahigh resolution mass data of natural organic matter. Anal. Chem. 79 (4), 1758-1763.

[3] Stenson, A. C.; Landing, W. M.; Marshall, A. G.; Cooper, W. T., 2002. Ionization and fragmentation of humic substances in electrospray ionization Fourier transform-ion cyclotron resonance mass spectrometry. Anal. Chem. 74 (17), 4397-4409.

[4] Riedel, T.; Biester, H.; Dittmar, T., 2012. Molecular fractionation of dissolved organic matter with metal salts. Environ. Sci. Technol. 46 (8), 4419-4426.
